# Autoantibodies neutralizing type I IFNs underlie severe tick-borne encephalitis in ∼10% of patients

**DOI:** 10.1084/jem.20240637

**Published:** 2024-09-24

**Authors:** Adrian Gervais, Astrid Marchal, Andrea Fortova, Michaela Berankova, Lenka Krbkova, Martina Pychova, Jiri Salat, Shuxiang Zhao, Nacim Kerrouche, Tom Le Voyer, Karin Stiasny, Simon Raffl, Anne Schieber Pachart, Samira Fafi-Kremer, Simon Gravier, Davide F. Robbiani, Laurent Abel, Margaret R. MacDonald, Charles M. Rice, Gaia Weissmann, Tarek Kamal Eldin, Eva Robatscher, Elke Maria Erne, Elisabetta Pagani, Alessandro Borghesi, Anne Puel, Paul Bastard, Aurélie Velay, Martin Martinot, Yves Hansmann, Judith H. Aberle, Daniel Ruzek, Aurélie Cobat, Shen-Ying Zhang, Jean-Laurent Casanova

**Affiliations:** 1 Laboratory of Human Genetics of Infectious Diseases, Necker Branch, Institut National de la Santé et de la Recherche Médicale (INSERM) U1163, Necker Hospital for Sick Children, Paris, France; 2 Imagine Institute, Paris Cité University, Paris, France; 3 Laboratory of Emerging Viral Diseases, Veterinary Research Institute, Brno, Czech Republic; 4 Department of Experimental Biology, Faculty of Science, Masaryk University, Brno, Czech Republic; 5 Institute of Parasitology, Biology Centre of the Czech Academy of Science, České Budějovice, Czech Republic; 6 Department of Children’s Infectious Diseases, University Hospital and Faculty of Medicine, Masaryk University, Brno, Czech Republic; 7 Department of Infectious Diseases, University Hospital Brno and Faculty of Medicine, Masaryk University, Brno, Czech Republic; 8 St. Giles Laboratory of Human Genetics of Infectious Diseases, Rockefeller Branch, Rockefeller University, New York, NY, USA; 9 Clinical Immunology Department, Assistance Publique Hôpitaux de Paris (AP-HP), Saint-Louis Hospital, Paris, France; 10 Medical University of Vienna, Center for Virology, Vienna, Austria; 11 Clinical Research Department, Hôpitaux Civils de Colmar, Colmar, France; 12 Institut de Virologie, Strasbourg University Hospital, Strasbourg University, INSERM Unité Mixte de Recherche (UMR) S1109, Strasbourg, France; 13 Infectious Diseases Department, Hôpitaux Civils, Colmar, France; 14 Institute for Research in Biomedicine, Università della Svizzera italiana, Bellinzona, Switzerland; 15 Laboratory of Virology and Infectious Disease, The Rockefeller University, New York, NY, USA; 16 Department of Pediatrics and Neonatology, F. Tappeiner Hospital, Merano, Italy; 17 Infectious Disease Unit, Provincial Hospital of Bolzano (SABES-ASDAA), Lehrkrankenhaus der Paracelsus Medizinischen Privatuniversität, Bolzano, Italy; 18 Laboratory of Microbiology and Virology, SABES-ASDAA, Lehrkrankenhaus der Paracelsus Medizinischen Privatuniversität, Bolzano, Italy; 19 Neonatal Intensive Care Unit, San Matteo Research Hospital, Pavia, Italy; 20 School of Life Sciences, Swiss Federal Institute of Technology, Lausanne, Switzerland; 21 Pediatric Hematology-Immunology and Rheumatology Unit, Necker Hospital for Sick Children, AP-HP, Paris, France; 22 CHU de Strasbourg, Service des Maladies Infectieuses et Tropicales, Strasbourg, France; 23 Howard Hughes Medical Institute, New York, NY, USA; 24 Department of Pediatrics, Necker Hospital for Sick Children, AP-HP, Paris, France

## Abstract

Tick-borne encephalitis (TBE) virus (TBEV) is transmitted to humans via tick bites. Infection is benign in >90% of the cases but can cause mild (<5%), moderate (<4%), or severe (<1%) encephalitis. We show here that ∼10% of patients hospitalized for severe TBE in cohorts from Austria, Czech Republic, and France carry auto-Abs neutralizing IFN-α2, -β, and/or -ω at the onset of disease, contrasting with only ∼1% of patients with moderate and mild TBE. These auto-Abs were found in two of eight patients who died and none of 13 with silent infection. The odds ratios (OR) for severe TBE in individuals with these auto-Abs relative to those without them in the general population were 4.9 (95% CI: 1.5–15.9, P < 0.0001) for the neutralization of only 100 pg/ml IFN-α2 and/or -ω, and 20.8 (95% CI: 4.5–97.4, P < 0.0001) for the neutralization of 10 ng/ml IFN-α2 and -ω. Auto-Abs neutralizing type I IFNs accounted for ∼10% of severe TBE cases in these three European cohorts.

## Teaser

Infection with tick-borne encephalitis (TBE) virus (TBEV) is usually benign but may cause severe encephalitis in rare cases. Pre-existing autoantibodies neutralizing type I IFNs underlie ∼10% of these severe cases, strongly increasing the risk of severe disease relative to the absence of these autoantibodies.

## Introduction

Tick-borne encephalitis virus (TBEV) is a human-tropic arthropod-borne virus from the Flaviviridae, the largest family of human arboviruses. TBEV is a single-stranded positive-sense RNA virus primarily transmitted to humans via the bite of infected ticks, typically *Ixodes ricinus* and *Ixodes persulcatus*, which serve as both vector and reservoir hosts of the European and Asian subtypes, respectively (Mansfield et al., 2009). More rarely, TBEV can be transmitted via unpasteurized milk or dairy products from cattle exposed to ticks in endemic areas (Elbaz et al., 2022). Around 10,000–12,000 clinical cases of tick-borne encephalitis (TBE) are diagnosed and reported worldwide annually (World Health Organization), including 3,000–4,000 cases in Europe (European Centre for Disease Prevention and Control). The incidence of TBE is increasing worldwide, with about 0.9 cases per 100,000 individuals in 2020 (Parfut et al., 2023), to the point that TBEV is now considered an emerging health threat in Europe and Asia, where it is endemic to many regions (Dobler, 2010; Dumpis et al., 1999; Kaiser, 2008; Lindquist and Vapalahti, 2008; Mansfield et al., 2009). Infections, like those with many other arboviruses, are typically silent (about 70–98% of cases). More rarely, they can generate clinical manifestations, ranging from self-healing fever to severe neurological illness (Dobler, 2010; Dumpis et al., 1999; Gustafson et al., 1992; Kaiser, 2008).

The proportion of silent cases is probably underestimated because many such infections remain undiagnosed. A recent serological study suggested that asymptomatic infections occur in 5.6% of the Swiss population (Ackermann-Gäumann et al., 2023). Symptomatic infections mostly present in two phases. During the initial phase (beginning 2–7 days after the bite), patients may develop non-specific symptoms, such as moderate fever, headache, body pain (myalgia and arthralgia), fatigue, general malaise, anorexia, and nausea (Lindquist and Vapalahti, 2008). These signs gradually abate in most patients, but a second phase of TBE occurs in ∼2–30% of cases, about 50% of whom suffer meningitis, ∼40% meningoencephalitis, and ∼5–10% meningoencephalomyelitis (Bogovic et al., 2010; Kaiser, 2008). Patients with TBE are typically hospitalized and receive supportive care based on the severity of clinical manifestations (Kaiser, 1999); 1–2% die and 10–20% of the survivors display neurological sequelae (Kaiser, 2012). There is no specific antiviral treatment for TBE but vaccination with inactivated viral strains is safe and effective. Nevertheless, vaccination coverage remains relatively low in several countries where TBEV is endemic (Pilz et al., 2023; Ruzek et al., 2019; Zavadska et al., 2013).

The cause and mechanism of TBE—which occurs in 2–30% of patients with diagnosed TBEV infection and probably in a much smaller proportion of infected individuals—remain unknown. TBEV-induced disease can occur in all age groups, but epidemiological studies have suggested that the risk is greater in older individuals (>55 years of age, with the highest proportion of severe cases among patients aged 70–79 years), as reported for other encephalitic orthoflaviviruses, including West Nile virus (WNV) (Kaiser, 2008; Radzišauskienė et al., 2020), and adverse reactions to the yellow fever virus (YFV) live-attenuated vaccines (Seligman, 2014). During initial viremia, TBEV may cross the blood–brain barrier and enter the central nervous system (CNS), where it targets primarily neurons (Schultze et al., 2007; Stone and Pinto, 2023). The infection of porcine embryonic kidney cells with TBEV activates innate immune signaling via engagement with the cytosolic double-stranded RNA–sensing RIG-I and MDA5, leading to the translocation of IFN regulatory factor 3 (IRF-3) to the nucleus, suggesting that type I IFNs are produced upon TBEV infection (Goonawardane et al., 2022). Interestingly, the NS5 protein of TBEV acts as an IFN antagonist, inhibiting the IFN-stimulated JAK–STAT pathway by blocking STAT1 phosphorylation, thereby inhibiting the expression of antiviral genes (Best et al., 2005; Werme et al., 2008). Moreover, TBEV can infect human and murine neurons and astrocytes without decreasing their survival, triggering the production of type I IFNs, which control the replication of many viruses in vitro (Bílý et al., 2015; Jorgačevski and Potokar, 2023; Lindqvist et al., 2018; Palus et al., 2014; Pokorna Formanova et al., 2019; Potokar et al., 2014).

No inborn errors of type I IFN immunity have ever been reported in patients with TBE, but this may reflect an absence of efforts to detect such conditions in these patients (Ignatieva et al., 2017; Schoggins et al., 2011; Yoshii, 2019). We recently reported the detection of autoantibodies (auto-Abs) neutralizing type I IFN-α and/or IFN-ω in about 40% of cases of WNV encephalitis (Gervais et al., 2023). WNV is an orthoflavivirus transmitted by mosquitoes (Smithburn et al., 1940). These auto-Abs also accounted for three of eight severe adverse reactions to subcutaneous inoculation with the YFV-17D live attenuated orthoflavivirus vaccine, including one patient with a neurological presentation (Bastard et al., 2021a). We and others have shown that such auto-Abs can underlie life-threatening cases of COVID-19 (Bastard et al., 2020, 2021a, 2021b, 2023, 2024b; Puel et al., 2022), influenza (Zhang et al., 2022), and Middle-East Respiratory Syndrome (MERS) pneumonia (Alotaibi et al., 2023). These auto-Abs are present before these viral infections occur and are causal for severe disease (Bastard et al., 2024a; Hale, 2023). In this context, we hypothesized that circulating auto-Abs neutralizing type I IFNs might underlie severe forms of TBE, in at least some patients.

## Results and discussion

### Four cohorts of patients with TBEV infection

**Figure 1.**
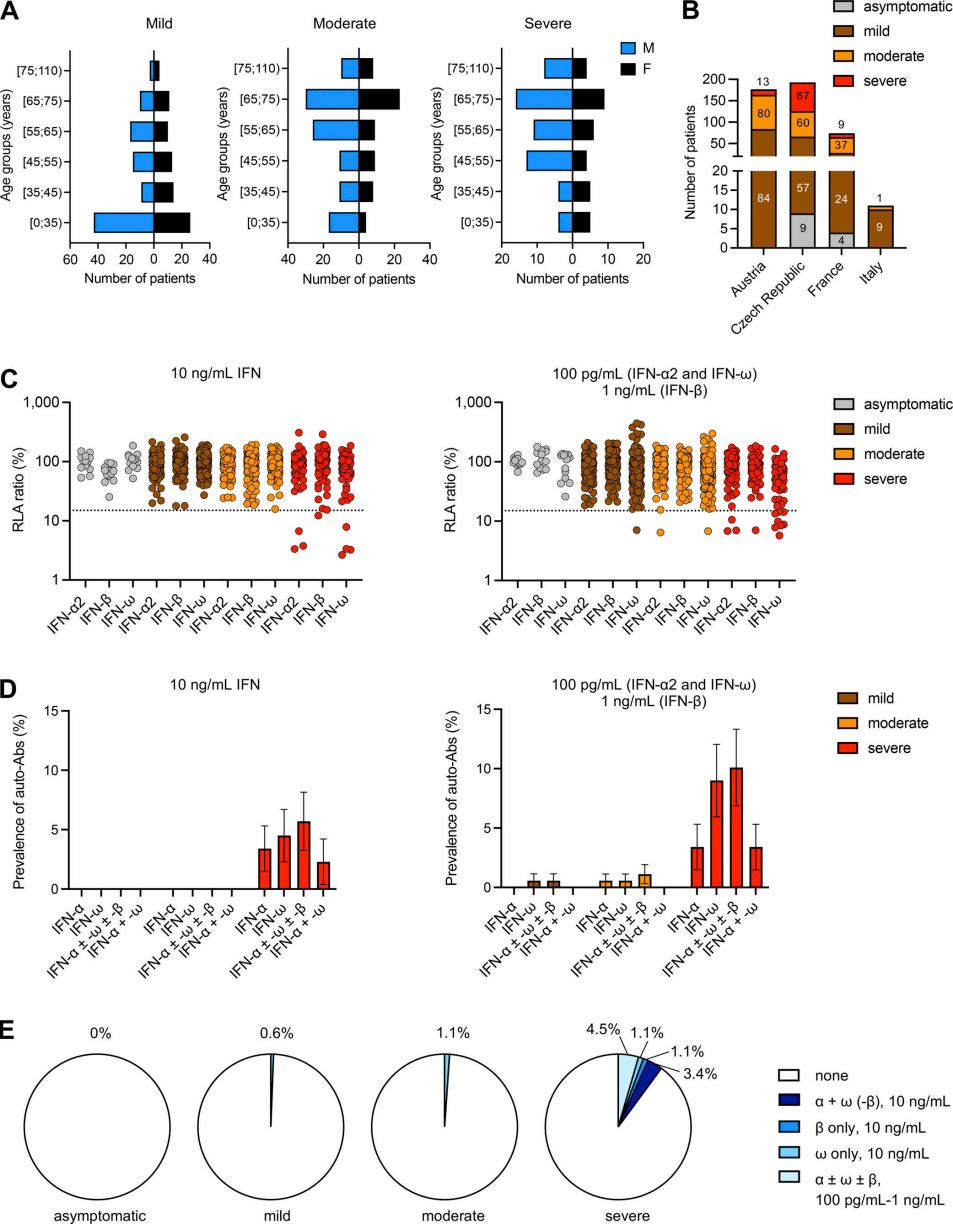
**Demographic and phenotypic distributions of the TBE cohort and auto-Abs neutralizing type I IFNs in individuals infected with TBEV. (A)** Age and sex distribution of the patients according to TBE severity. **(B)** Composition of the TBE cohort: number of individuals in each TBE group enrolled at each center. **(C)** Luciferase-based neutralization assay to detect auto-Abs neutralizing 10 ng/ml IFN-α2, IFN-ω, or IFN-β (left panel) and 100 pg/ml IFN-α2 or IFN-ω, or 1 ng/ml IFN-β (right panel). Plasma samples from asymptomatic TBE cases (gray), patients with mild TBE (brown), patients with moderate TBE (orange), and patients with severe TBE (red) were diluted 1:10. HEK293T cells were transfected with (1) a plasmid containing the firefly luciferase gene under the control of an ISRE-containing promotor and (2) a plasmid containing the *Renilla* luciferase gene. The cells were then treated with type I IFNs and RLA was calculated by normalizing firefly luciferase activity against *Renilla* luciferase activity. An RLA <15% of the median RLA for healthy controls was considered to correspond to neutralizing activity (dotted line; Bastard et al., 2021a). Each sample was tested once. **(D)** Proportions of individuals with auto-Abs neutralizing type I IFNs at a concentration of 10 ng/ml (left) or 1 ng/ml–100 pg/ml (right) in the three groups of TBE patients (mild, moderate, severe), as determined with the luciferase-based neutralization assay. IFN-α, auto-Abs neutralizing IFN-α2 (regardless of their effects on other IFNs); IFN-ω, auto-Abs neutralizing IFN-ω (regardless of their effects on other IFNs); IFN-α ± ω ± β, auto-Abs neutralizing IFN-α2 and/or IFN-ω and/or IFN-β; IFN-α + ω, auto-Abs neutralizing both IFN-α2 and IFN-ω. The bars indicate the upper and lower limits of the 95% CI. **(E)** Proportion of type I IFNs neutralized in the three groups of TBE patients (mild, moderate, severe) and in individuals with silent infection according to the nature and combination of auto-Abs.

We studied 441 TBEV-infected patients from Austria (177), the Czech Republic (184), France (70), and Italy (10). TBE was “mild,” defined as meningitis without CNS dysfunction or monofocal neurological signs (presentation as fever or headache), in 174 patients (84 Austrian, 57 Czech, 24 French, and 9 Italian); “moderate,” defined as encephalitis with moderate CNS dysfunction and/or monofocal neurological signs (including altered consciousness, ataxia, tremor, and dysphagia) in 178 patients (80 Austrian, 60 Czech, 37 French, and 1 Italian); and “severe,” defined as encephalitis with severe CNS dysfunction and/or multifocal neurological signs (including seizures, central paresis, bulbar symptoms, need for mechanical ventilation) in 89 patients (13 Austrian, 67 Czech, 9 French, and 0 Italian) (Fig. 1, A and B). All 441 patients required hospitalization. Three Czech patients (two mild cases and one moderate case), and one French patient (a moderate case) had been vaccinated against TBE. Another nine Czech and four French individuals with asymptomatic infection and high levels of anti-TBEV neutralizing antibodies detected during blood donation were included in the study. There was clear evidence of TBEV infection in all the individuals enrolled, as documented by the presence of TBEV-specific IgM and IgG antibodies in serum and/or TBEV-specific IgM antibodies in cerebrospinal fluid. The mean ages (standard deviation, SD) of patients with mild, moderate, and severe TBE were 41.7 (20.4), 56.6 (16.3), and 57.6 (16.5) years, respectively. The mean age (SD) at blood sampling for the nine asymptomatically infected blood donors was 40.3 (9.6) years; their age at infection was unknown. The proportion of men was 55% for mild, 66% for moderate, and 63% for severe TBE. Mortality was assessed based on vital status data. Eight deaths were reported, all in patients with severe TBE (9.0%) (Table S1). None of the 70 French patients died following TBE, whereas three patients from the Czech cohort and five from the Austrian cohort died. Interestingly, the severe TBE cases with auto-Abs were infected in 2011 (2/9), 2017 (1/9), or 2018 (6/9), suggesting that the disease may have been more severe in these years. Indeed, the epidemiology of TBE in affected countries is marked by strong annual variations in the number of hospitalized cases and fluctuations over time, and the mechanisms underlying this variation remain incomplete (Heinz et al., 2015). However, the fact that most of our cases were infected in 2011 and 2018 suggests that there may be a recruitment bias.

### Auto-Abs neutralizing IFN-α2, -β, and/or -ω in patients infected with TBEV

**Figure S1.**
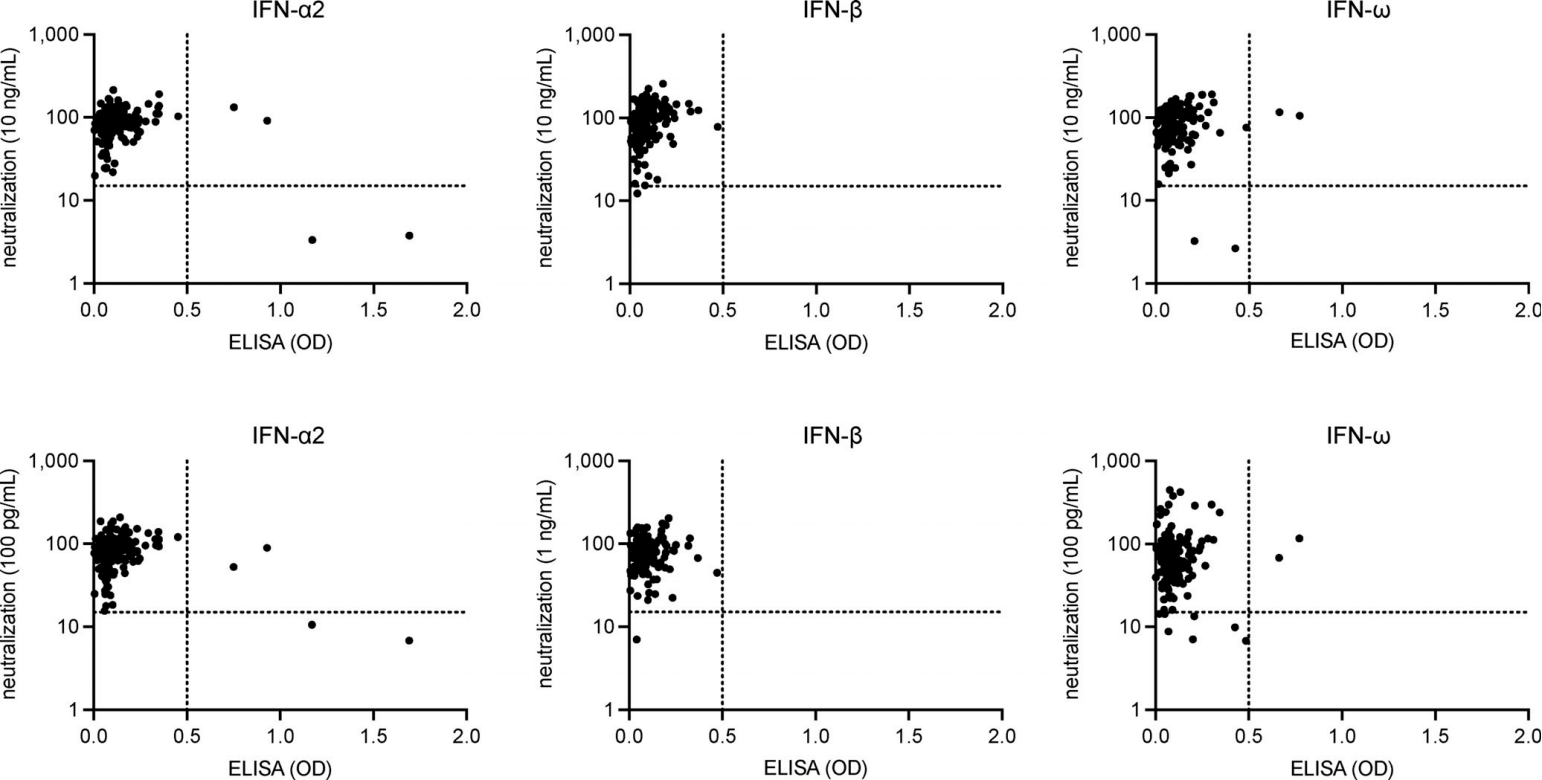
**Correlation between ELISA and neutralization assay results for the detection of auto-Abs neutralizing type I IFNs**.

Using a previously described luciferase-based neutralization assay (Bastard et al., 2021a), we tested 1:10 dilutions of serum or plasma from all enrolled subjects for the neutralization of high (10 ng/ml) or low (100 pg/ml) concentrations of non-glycosylated IFN-α2 and/or IFN-ω, and high (10 ng/ml) or intermediate (1 ng/ml) concentrations of glycosylated IFN-β. Most of the samples from symptomatic cases (383/441, 87%) were collected within 1 wk of the onset of neurological manifestations. The incubation period has been estimated at 7–14 days (Kaiser, 1999). None of the patients with mild or moderate TBE had auto-Abs neutralizing high concentrations of IFNs. By contrast, three patients with severe TBE had auto-Abs neutralizing both IFN-α2 and IFN-ω, one had auto-Abs neutralizing IFN-ω only, and another had auto-Abs neutralizing IFN-β only (five patients in total, 5.6% of severe cases) at high concentrations (Fig. 1, C and D). For lower concentrations, one patient with mild and another with moderate TBE had auto-Abs neutralizing IFN-ω only, another with moderate TBE had auto-Abs neutralizing IFN-α2 only, whereas three patients with severe TBE had auto-Abs neutralizing both IFN-α2 and IFN-ω, another had auto-Abs neutralizing IFN-β only, and five had auto-Abs neutralizing IFN-ω only (a total of nine severe patients, 10.1%) (Fig. 1, C and D; and Table S2). In the mild and moderate TBE groups, 0.6% (1/174) had auto-Abs neutralizing low concentrations of IFN-ω, and 1.1% (2/178) had auto-Abs neutralizing low concentrations of IFN-α2 or IFN-ω, respectively (Fig. 1 E and Table S2). The median ages (SD) of patients with severe TBE with and without auto-Abs were 61.0 (16.9) and 57.2 (16.5) years, respectively. None of the 13 asymptomatically infected individuals had auto-Abs against type I IFNs. Auto-Abs neutralizing IFN-α2 were detectable by ELISA, whereas those neutralizing IFN-β or low concentrations of IFN-ω were poorly detected, consistent with previous reports (Fig. S1) (Gervais et al., 2023). Two of the eight patients with severe TBE and auto-Abs died following infection, suggesting a higher risk of death among patients with severe TBE and auto-Abs (2/8, 25%) than in those with severe TBE without auto-Abs (6/80, 7.5%). Our findings also indicate that the neutralization of a single type I IFN can be sufficient to impair anti-TBEV immunity, consistent with previous findings in patients with COVID-19, influenza, and WNV encephalitis (Bastard et al., 2020, 2021a; Gervais et al., 2023; Zhang et al., 2022). In particular, auto-Abs against IFN-ω or IFN-β may underlie severe TBE. This is understandable as these two IFNs have the highest affinity for the type I IFN receptor. Overall, auto-Abs neutralizing IFNs were found in 10.1% of patients with severe TBE, whereas these auto-Abs were much less prevalent among patients with mild or moderate TBE and absent from individuals with silent infection.

### Prevalence of auto-Abs against type I IFNs in four cohorts

**Figure 2.**
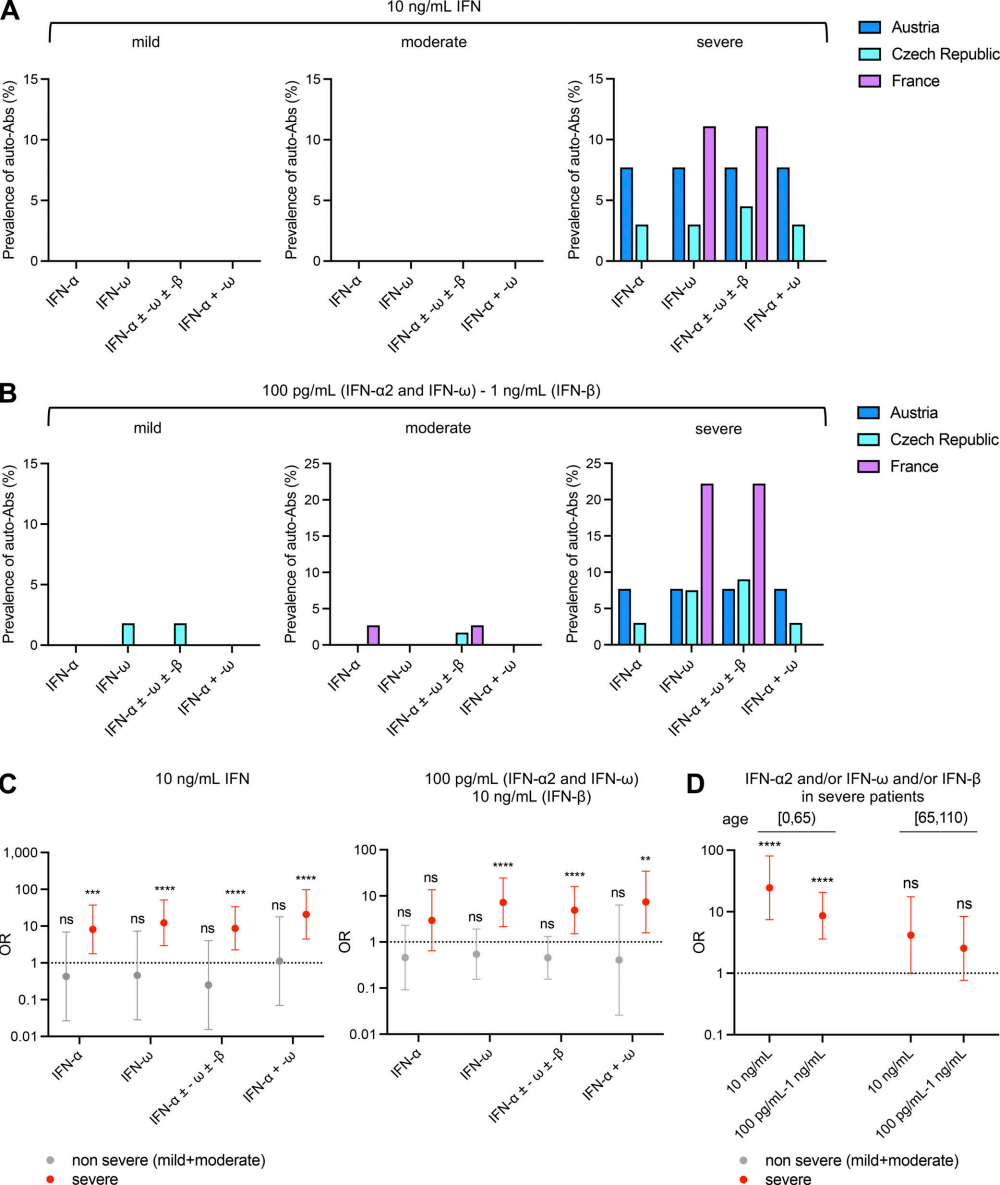
**Comparison of the proportions of individuals positive for auto-Abs neutralizing type I IFNs in the different TBE cohorts and estimation of the risk of severe TBE in auto-Ab-positive individuals relative to the general population. (A)** Comparison of auto-Ab prevalence in TBE patients from the Austrian, Czech, and French cohorts (considering auto-Abs neutralizing 10 ng/ml IFN). IFN-α, auto-Abs neutralizing IFN-α2 (regardless of their effects on other IFNs); IFN-ω, auto-Abs neutralizing IFN-ω (regardless of their effects on other IFNs); IFN-α ± ω ± β, auto-Abs neutralizing IFN-α2 and/or IFN-ω and/or IFN-β; IFN-α + ω, auto-Abs neutralizing both IFN-α2 and IFN-ω. **(B)** Comparison of auto-Ab prevalence in TBE patients from the Austrian, Czech, and French cohorts (considering auto-Abs neutralizing 100 pg/ml [IFN-α2 and IFN-ω] or 1 ng/ml [IFN-β]). IFN-α, auto-Abs neutralizing IFN-α2 (regardless of their effects on other IFNs); IFN-ω, auto-Abs neutralizing IFN-ω (regardless of their effects on other IFNs); IFN-α ± ω ± β, auto-Abs neutralizing IFN-α2 and/or IFN-ω and/or IFN-β; IFN-α + ω, auto-Abs neutralizing both IFN-α2 and IFN-ω. **(C)** OR for the presence of auto-Abs in individuals with non-severe (gray) or severe (red) TBE relative to the general population, with adjustment for age and sex by logistic regression. The horizontal bars indicate the upper and lower limits of the 95% CI. IFN-α, auto-Abs neutralizing IFN-α2 (regardless of their effects on other IFNs); IFN-ω, auto-Abs neutralizing IFN-ω (regardless of their effects on other IFNs); IFN-α ± ω ± β, auto-Abs neutralizing IFN-α2 and/or IFN-ω and/or IFN-β; IFN-α + ω, auto-Abs neutralizing both IFN-α2 and IFN-ω. **(D)** OR for the presence of auto-Abs in individuals with severe TBE relative to the general population by age group, with adjustment for sex by logistic regression. ORs were calculated separately for patients with severe TBE aged ≤65 and >65 years. The horizontal bars indicate the upper and lower limits of the 95% CI. IFN-α, auto-Abs neutralizing IFN-α2 (regardless of their effects on other IFNs); IFN-ω, auto-Abs neutralizing IFN-ω (regardless of their effects on other IFNs); IFN-α ± ω ± β, auto-Abs neutralizing IFN-α2 and/or IFN-ω and/or IFN-β; IFN-α + ω, auto-Abs neutralizing both IFN-α2 and IFN-ω. [0,65): individuals aged 0–65 years old; [65,110): individuals aged 65–110 years old; ns: non-significant; **P < 0.01, ***P < 0.001, ****P < 0.0001.

**Figure S2.**
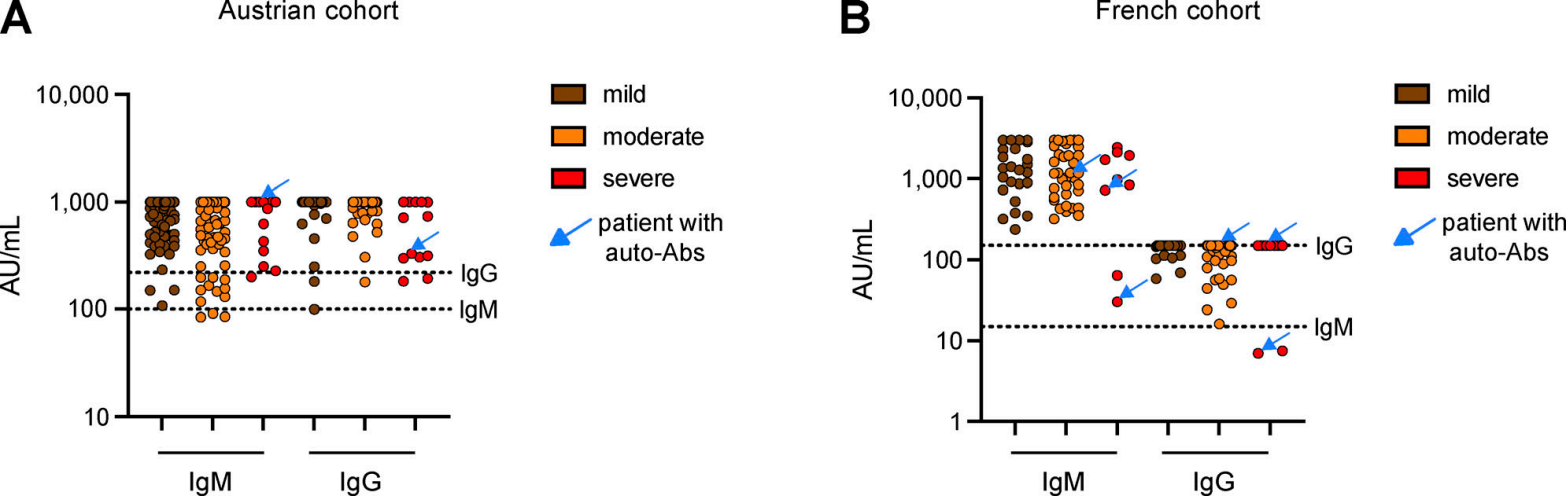
**Anti-TBEV IgG and IgM titers in the Austrian and French cohorts of TBE patients, by disease severity.** **(A and B)** The blue arrows show the patients with auto-Abs neutralizing type I IFNs. The dotted lines represent the thresholds for positivity. The assays used to detect anti-TBEV IgG and IgM titers differed between the two cohorts, accounting for the difference in positivity thresholds.

We compared the prevalence of auto-Abs neutralizing type I IFNs in the Austrian, Czech, French, and Italian cohorts. Age was similar in the four cohorts (51.6 [22.1] years in the Austrian cohort, 49.0 [17.9] years in the Czech cohort, 52.9 [15.7] years in the French cohort, and 44.9 [20.6] years in the Italian cohort). Among patients with severe TBE, the prevalence of auto-Abs neutralizing high concentrations of type I IFNs was ∼8% in the Austrian cohort, ∼5% in the Czech cohort, and ∼11% in the French cohort (Fig. 2, A and B). Auto-Abs neutralizing high concentrations of IFN-α2, IFN-β, and/or IFN-ω were found in the Austrian and Czech patients with severe TBE, whereas the higher prevalence of auto-Abs in the French cohort was driven exclusively by auto-Abs neutralizing IFN-ω (Fig. 2 A). This may reflect the smaller number of severe cases of TBE in the French cohort (nine), which may have been insufficient to capture the variety of auto-Abs observed in the Czech cohort. No Austrian or French patient with mild TBE had auto-Abs neutralizing lower concentrations of type I IFNs, whereas such auto-Abs were present in 1.8% or 1.7% of the Czech patients with mild TBE or moderate TBE, respectively, and in 2.7% of French patients with moderate TBE, but were absent from the moderate cases in the Austrian cohort (Fig. 2 B). All Italian TBE cases were mild or moderate, and all were negative for auto-Abs. For the neutralization of this lower concentration of IFNs, the prevalence of auto-Abs in patients with severe TBE was higher in the French cohort than in the Czech and Austrian cohorts (∼22%, ∼9%, and ∼8%, respectively) (Fig. 2 B). Part of these differences may be explained by the year or region of infection, as the epidemiology of TBE in affected countries is marked by strong annual variations in the number of hospitalized cases and fluctuations over time (Heinz et al., 2015), or by the sizes of the three cohorts. Anti-TBEV IgG and IgM titers were available for Austrian and French patients and did not seem to be correlated with disease severity or the presence of auto-Abs (Fig. S2). Overall, the prevalence of auto-Abs neutralizing type I IFNs was similarly low in patients with mild or moderate TBE from the four cohorts but high in patients with severe TBE (10.1%). The prevalence in patients with severe TBE in the French cohort was more than double that in the Austrian and Czech cohorts.

### Risk of severe tick-borne viral encephalitis in individuals with auto-Abs

We previously estimated the prevalence of neutralizing auto-Abs in the general population living in France by performing neutralization assays on samples from 34,159 healthy men and women aged 20–100 years (Bastard et al., 2021a). By comparing the proportions of TBE patients carrying auto-Abs with the proportions of individuals carrying the corresponding auto-Abs in the general population, we were able to estimate the risk of severe TBE conferred by the presence of these auto-Abs after adjustment for age and sex. We grouped the mild and moderate cases into a “non-severe” TBE group for this analysis. This group did not differ significantly from the general population in terms of auto-Ab prevalence for any of the combinations and concentrations of IFNs neutralized (Fig. 2 C and Table 1). By contrast, the prevalence of auto-Abs neutralizing IFNs was significantly higher in the severe TBE group than in the general population, except for auto-Abs neutralizing only 100 pg/ml IFN-α2 (Fig. 2 C and Table 1). At this concentration, the presence of auto-Abs neutralizing IFN-ω was associated with a higher risk of severe TBE (odds ratio [OR] = 7.2, 95% confidence interval [CI]: 2.1–24.4, P < 0.0001). Auto-Abs neutralizing at least one IFN and auto-Abs neutralizing both IFN-α2 and IFN-ω also conferred a higher risk of severe TBE (OR = 4.9, 95% CI: 1.5–15.9, P < 0.0001 and OR = 7.4, 95% CI: 1.6–34.3, P < 0.01, respectively). Auto-Abs neutralizing higher concentrations of IFNs (10 ng/ml) conferred a higher risk of severe TBE for all combinations of auto-Abs considered, with ORs ranging from 8.7 (95% CI: 2.3–33.7, P < 0.0001) for auto-Abs neutralizing at least one IFN to 20.8 (95% CI: 4.5–97.4, P < 0.0001) for auto-Abs neutralizing both IFN-α2 and IFN-ω. This suggests that auto-Abs neutralizing a higher concentration of IFNs, and auto-Abs neutralizing several IFNs are associated with more severe disease, consistent with our previous reports for auto-Abs neutralizing type I IFNs in other viral infections (Bastard et al., 2021a; Gervais et al., 2023; Zhang et al., 2022). The risk of severe TBE was higher in patients below the age of 65 years (OR = 25.8, 95% CI: 7.8–85.2, P < 0.0001 for auto-Abs neutralizing 10 ng/ml of at least one type I IFN) than in older patients (OR = 4.2, 95% CI: 0.98–17.5, P = 0.053) for the same combination of IFNs neutralized (Fig. 2 D). This finding is also consistent with previous reports of a greater impact of anti-type I IFN auto-Abs in individuals <70 years old with critical influenza pneumonia or severe WNV disease (Gervais et al., 2023; Zhang et al., 2022).

**Table 1.** Estimation of the risk of severe TBE in auto-Ab-positive individuals relative to the general population

Anti-type I IFN auto-Ab (amount of type I IFN neutralized, in plasma diluted 1:10)	OR (95% CI)	P value
Anti-IFN-α2 (10 ng/ml)	8.1 (1.8–37.4)	0.0005565
Anti-IFN-ω (10 ng/ml)	12.3 (3.0–51.4)	0.0000022
Anti-IFN-α2 (10 ng/ml) and/or anti-IFN-ω (10 ng/ml) and/or anti-IFN-β (10 ng/ml)	8.7 (2.3–33.7)	0.0000053
Anti-IFN-α2 (10 ng/ml) and anti-IFN-ω (10 ng/ml)	20.8 (4.5–97.4)	0.0000009
Anti-IFN-α2 (100 pg/ml)	2.9 (0.6–13.5)	0.0726972
Anti-IFN-ω (100 pg/ml)	7.2 (2.1–24.4)	0.0000002
Anti-IFN-α2 (100 pg/ml) and/or anti-IFN-ω (100 pg/ml) and/or anti-IFN-β (10 ng/ml)	4.9 (1.5–15.9)	0.0000113
Anti-IFN-α2 (100 pg/ml) and anti-IFN-ω (100 pg/ml)	7.4 (1.6–34.3)	0.0011799

### These auto-Abs neutralize the protective effect of type I IFNs against TBEV infection

**Figure 3.**
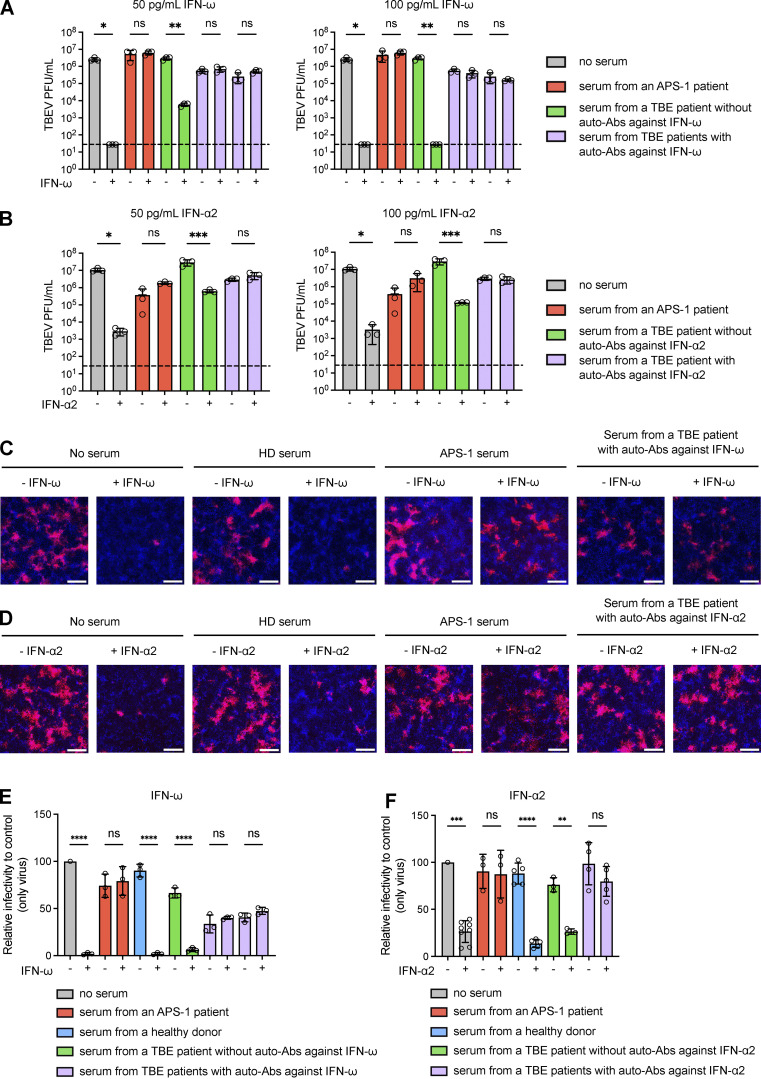
**TBEV infection and IFN treatment in Vero-E6 cells. (A and B)** Plaque assay showing TBEV viral titers (PFU/ml) in Vero-E6 cells left untreated or treated with serum samples from patients with or without auto-Abs neutralizing IFN-ω in the presence of 50 pg/ml IFN-ω (A, left panel), 100 pg/ml IFN-ω (A, right panel), 50 pg/ml IFN-α2 (B, left panel), or 100 pg/ml IFN-α2 (B, right panel). For each set of serum conditions, we compared the values obtained in the presence and absence of IFN treatment in an ordinary one-way ANOVA with Bonferroni correction (as implemented in GraphPad Prism version 10.2.3). ns: non-significant; *: P < 0.05; **: P < 0.01; ***: P < 0.001. The dashed line indicates the limit of detection of the plaque assay. **(C and D)** Fluorescence microscopy images of Vero-E6 cells with and without treatment with 50 pg/ml IFN-ω (C) or 50 pg/ml IFN-α2 (D) in the absence of serum or the presence of serum from a healthy donor (HD), an APS-1 patient (with auto-Abs against IFN-ω and IFN-α2), a TBE patient with auto-Abs against IFN-ω (C) or a TBE patient with auto-Abs against IFN-α2 (D). Vero-E6 cells were then infected with mCherry-TBEV. The nuclei were stained with Hoechst stain before the measurement of fluorescence. The scale bars represent 300 µm. **(E and F)** Quantification of TBEV-positive Vero-E6 cells normalized against the percentage of cells infected in TBEV-only conditions, for different serum samples, with or without IFN-ω (E) or IFN-α2 (F) treatment. ns: non-significant; **: P < 0.01; ***: P < 0.001; ****: P < 0.0001. For each set of serum conditions, the values obtained in the presence and absence of IFN treatment were compared in an ordinary one-way ANOVA with Bonferroni correction (as implemented in GraphPad Prism version 10.2.3).

We tested the hypothesis that auto-Abs against type I IFNs impair the protective antiviral functions of type I IFNs against TBEV infection using Vero-E6 cells as a surrogate cellular model in vitro. We incubated Vero-E6 cells with a mixture of IFN-α2 or IFN-ω (50 or 100 pg/ml) and serum samples from patients with auto-Abs against IFN-α2 (*n* = 1) or IFN-ω (*n* = 2) or healthy donor serum for 24 h. The medium was then replaced with fresh medium, and TBEV (strain Hypr) titers were determined 48 h after infection in a plaque assay. We found that Vero-E6 cells without serum or IFN-ω infected with TBEV had high TBEV titers, while these titers were much lower after treatment with IFN-ω (Fig. 3 A). Similar results were obtained when Vero-E6 cells were treated with serum from a TBE patient without auto-Abs against IFN-ω (Fig. 3 B). By contrast, Vero-E6 cells treated with serum from two TBE patients with auto-Abs against IFN-ω and infected with TBEV had high TBEV titers regardless of IFN-ω treatment, demonstrating that the auto-Abs of these patients neutralized the protective effect of IFN-ω against TBEV in vitro (Fig. 3 A). A similar result was obtained with serum from a patient with auto-Abs to IFN-α2 treated with IFN-α2 (Fig. 3 B). We also performed fluorescence microscopy on Vero-E6 cells incubated with serum from patients or healthy donors and IFN-α2 or IFN-ω and infected with TBEV, quantifying TBEV positivity in cells (Fig. 3, C–F). The percentage of positive cells for each set of conditions was normalized by dividing by the percentage of positive cells in the absence of serum. TBEV infection levels were high in Vero-E6 cells untreated with IFN-ω and incubated without serum or with healthy donor serum or serum from TBE patients without auto-Abs (75–100% of levels in the absence of serum), but this effect was fully rescued by preincubation with IFN-ω (0–8%) (Fig. 3 E). By contrast, Vero-E6 cells treated with serum from patients with auto-Abs against IFN-ω had similar infection levels regardless of their prior incubation with or without IFN-ω, as for control serum from an APS-1 patient with auto-Abs against IFN-ω, demonstrating the neutralizing effect of these auto-Abs against IFN-ω (Fig. 3 E). Similar results were obtained for the antiviral effect of IFN-α2 with serum samples from patients positive for auto-Abs against IFN-α2 (Fig. 3 F).

### Auto-Abs neutralizing type I IFNs are stable over time

**Figure 4.**
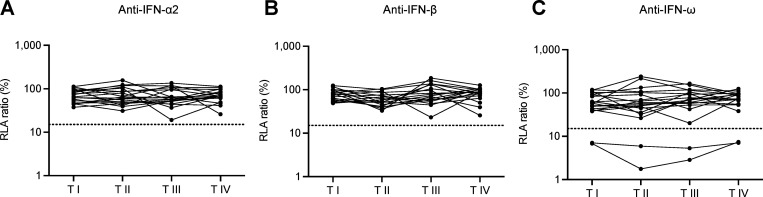
**Auto-Abs neutralizing type I IFNs in longitudinal samples. (A–C)** Auto-Abs neutralizing IFN-α2 (A), IFN-ω (B), and IFN-β (C), as determined with the luciferase-based neutralization assay, at different time points after infection. Each sample was tested once with an IFN concentration of 100 pg/ml (IFN-α2 and IFN-ω) or 1 ng/ml (IFN-β). I: time of admission to hospital, II: 2 days after admission, III: hospital discharge, and IV: follow-up (1–3 mo).

Finally, we studied longitudinal serum or plasma samples for 19 Czech patients (6 patients with mild, 12 with moderate, and 1 with severe TBE) obtained at the time of admission to the hospital (I), 2 days after admission (II), at discharge (III), and during follow-up (1–3 mo) (IV). We tested these samples for auto-Abs neutralizing type I IFNs. None of the available samples displayed neutralizing activity toward IFN-α2 or IFN-β at any of the timepoints available. Two samples from two patients (one with moderate and one with severe TBE) neutralized 100 pg/ml IFN-ω at all the available time points (Fig. 4, A–C). This result is consistent with previous studies reporting sustained type I IFN–neutralizing activity over time in longitudinal samples from patients with viral infections or genetic conditions underlying the production of auto-Abs neutralizing type I IFNs (Shaw et al., 2022; Sonja et al., 2024, *Preprint*; Su et al., 2023). However, it contrasts with other studies reporting some fluctuation over time (Chang et al., 2021; Shaw et al., 2022; Steels et al., 2022). These data suggest that the auto-Abs against type I IFN were not triggered by TBEV infection and that they were not transient, consistent with our previous findings for patients with other viral infections (Gervais et al., 2023; Le Voyer et al., 2023).

Overall, about 10% of patients hospitalized for severe TBE in three European cohorts—in Austria, the Czech Republic, and France—were found to carry auto-Abs neutralizing type I IFNs at admission. This detection of auto-Abs in a significant proportion of patients with severe WNV encephalitis and then in TBE suggests that germline genetic deficiencies of type I IFN immunity should be sought in patients with either of these forms of viral encephalitis without auto-Abs against type I IFNs. Our findings also suggest that people at risk of producing such auto-Abs—including patients with a history of severe viral disease, autoimmunity, an inborn error of tolerance to self, and elderly individuals—may benefit from being tested if they inhabit or plan to travel to an area in which TBEV is endemic (Bastard et al., 2021a; Groen et al., 2024, *Preprint*). This is particularly important given the existence of both an effective TBE vaccine and the absence of a specific antiviral therapy for TBE, which is a serious illness requiring hospitalization. Even patients with mild or moderate TBE remain ill for weeks. Moreover, the outcome of severe TBE is poor, with a high risk of death or sequelae (Kaiser, 2008, 2012; Ruzek et al., 2019). Our findings finally suggest that treatment with a type I IFN, such as IFN-α2 or IFN-β, may be beneficial in patients hospitalized for TBE, with or without auto-Abs. Patients with auto-Abs neutralizing IFN-α2 may benefit from treatment with IFN-β, whereas those with auto-Abs neutralizing IFN-β may benefit from treatment with IFN-α2. We recently developed a rapid diagnostic test that can provide results within a few hours (Gervais et al., 2024). The use of this test at admission is warranted for patients with suspected TBE.

## Materials and methods

### Patients

We enrolled an international cohort of 454 individuals aged 3–89 years with documented TBEV infection, 61.3% of whom were men and 38.7% women, from Austria, the Czech Republic, France, and Italy. TBEV infection was diagnosed on the basis of a serological demonstration of the presence of TBEV-specific IgM with or without the concomitant presence of specific IgG antibodies in serum, or TBEV-specific IgM antibodies in cerebrospinal fluid during acute infection. Individuals were classified according to the presence and/or severity of clinical manifestations, as previously defined (Agudelo et al., 2021; Bogovic and Strle, 2015; Ruzek et al., 2019). Briefly, disease severity was evaluated as follows: mild = flu-like symptoms with meningeal irritation defined as meningitis, characterized by fever, fatigue, nausea, headache, back pain, arthralgia/myalgia, and neck or back stiffness; moderate = similar to mild, but with tremor, vertigo, somnolence, and photophobia, defined as meningoencephalitis; severe = prolonged neurological consequences, including ataxia, titubation, altered mental status, memory loss, quantitative disturbance of consciousness, and palsy, revealed as encephalitis, encephalomyelitis, or encephalomyeloradiculitis. Clinical data were obtained from the treating hospital. The cases of asymptomatic TBEV infection were blood donors with documented TBEV infection diagnosed during the screening of a donated blood sample who remained asymptomatic during follow-up. The study was performed in accordance with the Declaration of Helsinki. For the Austrian cohort, we analyzed anonymized leftover samples from antibody testing stored in a biobank in accordance with established protocols. The ethics committee of the Medical University of Vienna approved the study protocol (EK1291/2021). The Ethics Committee of the University Hospital in Brno (for Czech patients) approved the study. The Ethics Committee of the Provincial Hospital of Bolzano for Italian patients) (SABES-ASDAA), Lehrkrankenhaus der Paracelsus Medizinischen Privatuniversität, Bolzano, Italy, approved the study. All patients or their parents agreed to participate in the study and signed an informed consent form. The nine Czech individuals with asymptomatic TBEV infection were identified during the screening of 263 healthy blood donors who had not been vaccinated against TBE and had never had TBE. These results suggest that the prevalence of silent TBEV infections in the Czech general population is 3.4%, consistent with the results reported for Switzerland (Ackermann-Gäumann et al., 2023). The four French individuals with asymptomatic TBEV infection were identified during blood donation at the French Blood Bank.

### ELISA for detecting auto-Abs against type I IFNs

ELISA was performed as previously described (Puel et al., 2008). In brief, 96-well ELISA plates (MaxiSorp; Thermo Fisher Scientific) were coated by overnight incubation at 4°C with 1 μg/ml rhIFN-α (ref. number 130-108-984; Miltenyi Biotec), rhIFN-ω (ref. number 300-02J; Peprotech), or rhIFN-β (ref. number 300-02BC; Peprotech). The plates were washed (PBS/0.005% Tween), blocked by incubation with the same buffer supplemented with 2% BSA, washed, and incubated with 1:50 dilutions of plasma samples from the patients or controls for 2 h at room temperature (or with specific mAbs as positive controls). Each sample was tested once. Plates were thoroughly washed (PBS/0.005% Tween). Horseradish peroxidase (HRP)–conjugated Fc-specific IgG fractions from polyclonal goat antiserum against human IgG (Nordic Immunological Laboratories) were added to a final concentration of 1 μg/ml. Plates were incubated for 1 h at room temperature and washed. The substrate was added, and the optical density (OD) was measured at 450 and 540 nm. The final OD was calculated as follows: OD_450 nm_ – OD_540 nm_. All the incubation steps were performed with gentle shaking (600 rpm).

### Luciferase reporter assay

The blocking activity of anti-IFN-α2, anti-IFN-ω, and anti-IFN-β auto-Abs was determined with a reporter luciferase assay, as previously described (Bastard et al., 2021a). Briefly, HEK293T cells were transfected with a plasmid encoding the firefly luciferase gene under the control of the human IFN-sensitive response element (ISRE) promoter in the pGL4.45 backbone and a plasmid constitutively expressing the *Renilla* luciferase as a control for transfection (pRL-SV40). Cells were transfected in the presence of the X-tremeGene9 transfection reagent (ref. number 6365779001; Sigma-Aldrich). After 24 h, cells in Dulbecco’s modified Eagle medium (DMEM; Thermo Fisher Scientific) supplemented with 2% fetal calf serum (FCS) and 10% healthy control or patient serum/plasma (after heat inactivation at 56°C, for 20 min) were either left unstimulated or were stimulated with unglycosylated rhIFN-α2 (ref. number 130-108-984; Miltenyi Biotec), unglycosylated rhIFN-ω (ref. number 300-02J; Peprotech) at a concentration of 10 ng/ml or 100 pg/ml, or glycosylated rhIFN-β (ref. number 300-02BC; Peprotech) at a concentration of 10 or 1 ng/ml for 16 h at 37°C under an atmosphere containing 5% CO_2_. Finally, the cells were lysed by incubation with lysis buffer (provided in the luciferase kit) for 20 min at room temperature and luciferase levels were measured with the Dual-Luciferase Reporter 1000 assay system (ref. number E1980; Promega) according to the manufacturer’s protocol. Luminescence intensity was measured with a VICTOR-X Multilabel Plate Reader (PerkinElmer Life Sciences). Firefly luciferase activity values were normalized against *Renilla* luciferase activity values. The resulting values (luciferase induction) were then normalized against the median level of induction for non-neutralizing samples and expressed as a percentage (relative luciferase activity [RLA] ratio, %). Samples were considered to be neutralizing if the RLA ratio was below 15% of the median value for controls tested on the same day.

### TBEV infection and IFN treatment in Vero-E6 cells

The ability of serum samples from patients to neutralize IFN-α2 and IFN-ω was assessed with Vero-E6 cells, as previously described (Gervais et al., 2023). Vero-E6 cells were used to seed TPP 96-well plates (Biotech) at a density of 3 × 10^4^ cells per well in DMEM supplemented with 10% FBS (ref. number FB-1001/100; Biosera) and 1% antibiotic/antimycotic solution (ref. number AAS-B/2; Capricorn Scientific). The following day, serum samples from the patients were incubated with 100 or 50 pg/ml recombinant IFN-α2 (M6041; Merck) or IFN-ω (SRP3061; Sigma-Aldrich) for 1 h at 37°C. A virus control (no serum, no IFN), a cell control (serum plus IFN), and a healthy donor serum control (without IFN) were included. The culture medium was removed from the cells and replaced with a mixture of serum and IFN. The plates were incubated for 24 h, the medium containing the serum and IFN mixture was removed, and the plates were washed once with PBS. Cells were then infected with TBEV at an MOI of 0.1 and incubated for 2 h at 37°C. The plates were washed once with PBS and fresh DMEM was added to the wells. After 48 h, the medium was collected and TBEV titers were determined in a plaque assay.

### Plaque assay

Virus titers were measured with a monolayer of A549 lung carcinoma cells. We prepared 10-fold dilutions of TBEV-infected medium in a 24-well tissue culture plate to which we added a suspension of A549 cells. After incubation for 4 h, we added 1.5% (wt/vol) carboxymethylcellulose in DMEM supplemented with 10% FBS (ref. number FB-1001/100; Biosera) and 1% antibiotic/antimycotic solution (ref. number AAS-B/2; Capricorn Scientific). After 5 days of incubation at 37°C under an atmosphere containing 5% CO_2_, the infected plates were washed with PBS and the cell monolayers were stained with naphthalene black. TBEV viral titers were calculated and are expressed in PFU per mL.

### Visualization by fluorescence microscopy and quantification of IFN neutralization

Vero-E6 cells were plated in 96-well µCLEAR BLACK CELLSTAR tissue culture plates (Grenier, Bio-One) at a density of 2 × 10^4^ cells per well. The cells were treated as described above, with the following modifications: serum samples were incubated with 50 pg/ml recombinant IFN-α2 or IFN-ω, the cells were infected with mCherry-TBEV (Haviernik et al., 2021) at an MOI of 1. Fluorescence microscopy was performed 120 h after infection. For quantification, cell nuclei were stained with Hoechst 33342 (Invitrogen) diluted 1:5,000 in PBS, which was used to replace the medium in the 96-well plate. The plate was then incubated at 37°C for 30 min. The ImageXpress Pico automatic imaging system and CellReporterXpress software (Molecular Devices) were used for image acquisition and analysis. Cell scoring was performed for each well, with three independent rectangular regions per cell analyzed. The percentage of the Vero-E6 cells positive for TBEV was calculated as a mean value for these three regions across replicates. Relative infectivity was determined by normalizing the percentage of TBEV-positive cells in each set of conditions relative to the control (virus only). Microscopy images were processed with ImageJ (version 1.54d).

### Anti-TBEV antibody ELISA

For the Austrian cohort, TBEV-specific antibody titers were determined as previously described (Stiasny et al., 2012). Briefly, TBEV-specific IgM antibodies were analyzed by IgM capture ELISA, and TBEV-specific IgG antibodies were analyzed in a three-layer ELISA. Titers were determined in arbitrary units (AU) relative to a standard polyclonal human anti-TBEV serum with a titer set to 1,000 AU. Twofold serial dilution curves of the standard were fitted by four-parameter logistic regression. The cutoffs for IgM (100 AU) and IgG (220 AU) antibody titers were defined on the basis of results for 45 and 90 orthoflavivirus negative sera, respectively. For the French cohort, all samples were tested for TBEV infection with commercial assays (SERION ELISA classic TBE Virus IgG/IgM; Institut Virion/Serion GmbH, Würzburg, Germany), the results of which were interpreted according to the manufacturer’s instructions (Septfons et al., 2023). Results are expressed in U/ml, and the positive cutoff values were 15 U/ml for TBEV IgM and 150 U/ml for TBEV IgG. French patients tested for anti-TBEV IgM and IgG were questioned about their history of tick bites, recent or past travel, and the geographic area concerned, and their history of vaccination for yellow fever, Japanese encephalitis, and TBE. If necessary, crossreactivity with other orthoflavivirus infections was excluded by performing a serum neutralization test (strain Hypr, GenBank ID U39292.1) on IgM- and IgG-positive samples for the purposes of confirmation (Beck et al., 2015). A serum sample was considered positive for TBEV if the cells were protected at a serum dilution of at least 1:20.

### Statistical analysis

ORs and P values for the effect of auto-Abs neutralizing each type I IFN in patients with severe TBE were estimated relative to healthy individuals from the general population and adjusted for age in three categories (≤40, 40–65, >65 years) and sex by logistic regression analysis with the glm function of R software. ORs and P values for the effect of auto-Abs on patients with non-severe TBE relative to healthy individuals were estimated and adjusted for age and sex with Firth’s bias-corrected logistic regression, as implemented in the logistf package of R software, due to the absence of auto-Ab carriers for some types of IFN. Where relevant, statistical test results are indicated in the corresponding figures. ns: not significant, *P < 0.05, **P < 0.01, ***P < 0.001, ****P < 0.0001. In the plaque assays and in assays quantifying TBEV-positive Vero-E6 cells in each set of serum conditions, we compared the values obtained in the presence and absence of IFN treatment in an ordinary one-way ANOVA with Bonferroni correction (as implemented in GraphPad Prism version 10.2.3).

### Online supplemental material

Fig. S1 provides additional data on the correlation between ELISA and neutralization assay results for the detection of auto-Abs neutralizing type I IFNs. Fig. S2 provides additional data on anti-TBEV IgG and IgM titers by disease severity level in the Austrian and French cohorts of TBE patients. The blue arrows show the patients with auto-Abs neutralizing type I IFNs. The dotted lines represent the thresholds for positivity. The assays used to detect anti-TBEV IgG and IgM titers differed between the two cohorts, accounting for the difference in positivity thresholds. Table S1 shows the demographic and clinical characteristics of the four cohorts of TBE patients studied. Table S2 shows general characteristic of TBE patients with auto-Abs neutralizing type I IFNs.

## Supplementary Material

Table S1 shows the demographic and clinical characteristics of the four cohorts of TBE patients studied.

Table S2 shows the general characteristic of TBE patients with auto-Abs neutralizing type I IFNs.

## Data Availability

All data supporting the findings of this study are available within the main text and supplemental material and from the corresponding authors upon request.
