# Supplementary material for: Autoantibodies neutralizing type I IFNs underlie severe tick-borne encephalitis in ∼10% of patients
Source: J Exp Med. 2024 Sep 24;221(10):e20240637. doi: 10.1084/jem.20240637 (PMC11448868; doi:10.1084/jem.20240637)
Supplement: Table S1 — shows the demographic and clinical characteristics of the four cohorts of TBE patients studied. [file JEM_20240637_TableS1.docx]

**Table S1: Demographic and clinical characteristics of the four cohorts of TBE patients studied**

|  | **Asymptomatic (*n*=13)** | **Mild TBE (*n*=174)** | **Moderate TBE (*n*=178)** | **Severe TBE (*n*=89)** |
| --- | --- | --- | --- | --- |
| **Mean age [SD] (years)** | 40.3 [9.6] | 41.7 [20.4] | 56.6 [16.3] | 57.6 [16.5] |
| **Sex** |  |  |  |  |
| F | 4 (30.7%) | 78 (44.8%) | 61 (34.3%) | 33 (37.1%) |
| M | 9 (69.3) | 96 (55.2%) | 117 (65.7%) | 56 (62.9) |
| **Mortality** | 0 | 0 | 0 | 8 (9.0%) |
| **Recruitment center** |  |  |  |  |
| Czech Republic | 13 (69.2%) | 57 (32.7%) | 60 (33.7%) | 67 (75.3%) |
| Austria | 0 | 84 (48.3%) | 80 (44.9%) | 13 (14.6%) |
| France | 4 (30.8%) | 24 (13.8%) | 37 (20.8%) | 9 (10.1%) |
| Italy | 0 | 9 (5.2%) | 1 (0.6%) | 0 |
| **Year of infection** |  |  |  |  |
| Unknown | 13 (100%) | 0 | 0 | 0 |
| 2011 | 0 | 13 (7.5%) | 8 (4.5%) | 25 (28.1%) |
| 2012 | 0 | 0 | 0 | 1 (1.1%) |
| 2013 | 0 | 2 (1.1%) | 0 | 0 |
| 2016 | 0 | 4 (2.3%) | 9 (5.1%) | 1 (1.1%) |
| 2017 | 0 | 4 (2.3%) | 4 (2.2%) | 2 (2.2%) |
| 2018 | 0 | 24 (13.8%) | 25 (14.0%) | 45 (50.6%) |
| 2019 | 0 | 8 (4.6%) | 5 (2.8%) | 3 (3.4%) |
| 2020 | 0 | 17 (9.8%) | 23 (12.9%) | 9 (10.1%) |
| 2021 | 0 | 34 (19.5%) | 26 (14.6%) | 0 |
| 2022 | 0 | 41 (23.6%) | 47 (26.4%) | 2 (2.2%) |
| 2023 | 0 | 27 (15.5%) | 31 (17.4%) | 1 (1.1%) |
