# Supplementary material for: Autoantibodies neutralizing type I IFNs underlie severe tick-borne encephalitis in ∼10% of patients
Source: J Exp Med. 2024 Sep 24;221(10):e20240637. doi: 10.1084/jem.20240637 (PMC11448868; doi:10.1084/jem.20240637)
Supplement: Table S2 — shows the general characteristic of TBE patients with auto-Abs neutralizing type I IFNs. [file JEM_20240637_TableS2.docx]

**Table S2: General characteristic of TBE patients with auto-Abs neutralizing type I IFNs**

| **TBE severity** | **Year of infection** | **Age** | **Sex** | **Survival** | **Country of origin** | **Type I IFN(s) neutralized** |
| --- | --- | --- | --- | --- | --- | --- |
| severe | 2018 | 65 | male | no | Austria | IFN-α2 and IFN-ω, high concentrations |
| mild | 2020 | 31 | male | yes | Czech Republic | IFN-ω, low concentrations |
| moderate | 2020 | 50 | female | yes | Czech Republic | IFN-ω, low concentrations |
| severe | 2011 | 74 | male | yes | Czech Republic | IFN-ω, low concentrations |
| severe | 2011 | 35 | female | yes | Czech Republic | IFN-ω, low concentrations |
| severe | 2018 | 49 | male | yes | Czech Republic | IFN-ω, low concentrations |
| severe | 2018 | 84 | male | no | Czech Republic | IFN-α2 and IFN-ω, high concentrations |
| severe | 2018 | 40 | male | yes | Czech Republic | IFN-β , high concentrations |
| severe | 2018 | 78 | female | yes | Czech Republic | IFN-α2 and IFN-ω, high concentrations |
| severe | 2017 | 60 | male | yes | France | IFN-ω, low concentrations |
| severe | 2018 | 64 | male | yes | France | IFN-ω, high concentrations |
| moderate | 2018 | 63 | male | yes | France | IFN-α2, low concentrations |
